# A nonet of novel species of *Monanthotaxis* (Annonaceae) from around Africa

**DOI:** 10.3897/phytokeys.69.9292

**Published:** 2016-08-30

**Authors:** Paul H. Hoekstra, Jan J. Wieringa, Lars W. Chatrou

**Affiliations:** 1 Naturalis Biodiversity Center (Section NHN), Herbarium Vadense, Darwinweg 2, 2333 CR Leiden, The Netherlands; 2 Biosystematics group, Wageningen University and Research centre, Droevendaalsesteeg 1, 6708 PB Wageningen, The Netherlands

**Keywords:** Monanthotaxis, Annonaceae, Africa, Gilbertiella, new species, Mayotte, Comoros, Gabon, Cameroon, Tanzania, Mozambique, Ivory Coast, Ghana, South Africa, Republic of Congo, Atewa Range, Ottotomo, Rondo

## Abstract

As part of an ongoing revision of the genus *Monanthotaxis* Baill. (Annonaceae), nine new species are described and one variety is reinstated to species rank. Two new species from West Africa (*Monanthotaxis aquila* P.H.Hoekstra, **sp. nov.** and *Monanthotaxis atewensis* P.H.Hoekstra, **sp. nov.**), four new species from Central Africa (*Monanthotaxis couvreurii* P.H.Hoekstra, **sp. nov.**, *Monanthotaxis latistamina* P.H.Hoekstra, **sp. nov.**, *Monanthotaxis tripetala* P.H.Hoekstra, **sp. nov.** and *Monanthotaxis zenkeri* P.H.Hoekstra, **sp. nov.**), one new species from Tanzania (*Monanthotaxis filipes* P.H.Hoekstra, **sp. nov.**), one new species from the area around Maputo (*Monanthotaxis maputensis* P.H.Hoekstra, **sp. nov.**), one new species from the Comoro Islands (*Monanthotaxis komorensis* P.H.Hoekstra, **sp. nov.**) and Monanthotaxis klainei (Engl.) Verdc. var. angustifolia (Boutique) Verdc. is raised to species level leading to the replacement name *Monanthotaxis atopostema* P.H.Hoekstra, **nom. nov.** (not *Monanthotaxis angustifolia* (Exell) Verdc.). Complete descriptions, comparisons with related species, ecological information and IUCN conservation assessments are given for the new species. Five species were classified as critical endangered, two species as endangered, one as vulnerable and one as least concern, warranting the need of further collecting and studying those species.

## Introduction

The genus *Monanthotaxis* Baill. belongs to the tribe Uvariae in the family Annonaceae (Chatrou et al. 2012). Species of *Monanthotaxis* are scandent shrubs or lianas, and are confined to tropical Africa and Madagascar. The generic circumscription of the genus has seen considerable changes in the past. The genus was described by Baillon (1890) based on the presence of a single whorl of six petals, and a single whorl of stamens. Most species of Annonaceae typically have two whorls of three petals, and many whorls of stamens. Baillon (1890) named the genus *Monanthotaxis* after these characteristics (in Greek mono = one, anthir = stamen, taxis = order). In the following 60 years three additional species were described in the genus *Monanthotaxis* that displayed these generic characteristics. Then, Verdcourt (1971) synonymized the genus *Enneastemon* Exell and the African species of the genus *Popowia* Endl. into *Monanthotaxis* based on their similarity. Species of *Popowia* only differed from *Monanthotaxis* by having the petals in two whorls, however species of *Enneastemon* have the petals intermediate between *Popowia* and *Monanthotaxis*, which is a single whorl at the base of the flower, and in two whorls apically. With the rise of molecular phylogenetic analyses it has appeared that the genus *Friesodielsia* is polyphyletic, with most of the African species being sister to the genus *Monanthotaxis* (Chatrou et al. 2012; Richardson et al. 2004; Wang et al. 2012). Further data sampling has also revealed that the monotypic genus *Exellia* is nested in *Monanthotaxis*.

Alongside a molecular phylogenetic analysis of *Monanthotaxis* and related genera, a taxonomic revision of the genus is being undertaken. This revision has revealed nine new species of the genus *Monanthotaxis* which are described in this article. Two are from West Africa, four from western Central Africa, one from Tanzania, one from Southern Mozambique and one from the Comoros. This follows a general pattern in recent revisions of both Annonaceae and other tropical African forest taxa that most new species are found in western central Africa and Tanzania (e.g. Bissiengou et al. 2013; Breteler and Wieringa 2008; Breteler 2010; Couvreur et al. 2006; van der Burgt et al. 2015; Wieringa and Mackinder 2012). Also Madagascar is an area with many undescribed species, this is also true for *Monanthotaxis*, at least seven species will be described in another paper. It is striking that all species here described (except *Monanthotaxis filipes*) had already been collected at least 40 year ago, some even more than 100 years ago. Although of many of them only recently good flowering material became available, this does prove the importance of herbaria, and the need for exploring their collections (Bebber et al. 2010). Each of these species belongs clearly to the genus *Monanthotaxis*, as they share the following morphological characteristics with all other species: a climbing habit, glaucous leaves, loosely coherent floral chambers, and moniliform monocarps. DNA sequences have confirmed their phylogenetic position within *Monanthotaxis*, and these analyses will be published soon in a separate paper with the new generic delimitation of *Monanthotaxis* (Guo et al. in press). With the species described here, the current number of species of *Monanthotaxis* will raise to 67.

## Material and methods

Over 2000 collections of *Monanthotaxis*, *Exellia*, *Gilbertiella* and African *Friesodielsia* were examined from the following herbaria: A, AMD, B, BM, BNRH, BR, BRLU, C, E, EA, FHO, G, GC, K, L, LBV, LISC, LISU, M, MA, MO, NU, NY, P, SRGH, U, US, WAG and YA. All measurements were taken from dried specimens, colours were described based on the collector’s information. For the species description the same terminology is being applied as in Hoekstra et al. (2014) with the exception that sterile stamens are called staminodes and the peduncle a sympodial rachis, to be in concordance with other Annonaceae literature (e.g. Endress and Armstrong 2011; Maas et al. 2003). The extent of occurrence and area of occupancy were calculated using GeoCAT (Bachman et al. 2011) and preliminary conservation status are proposed following the IUCN Red List Category Criteria (IUCN Standards and Petitions Subcommittee 2016).

## Taxonomic treatment

### Monanthotaxis atopostema

Taxon classificationPlantaeMagnolialesAnnonaceae

P.H.Hoekstra nom. nov.

urn:lsid:ipni.org:names:77157214-1

Monanthotaxis atopostema Replacement name for: Atopostema angustifolia Boutique, Bull. Jard. Bot. Brux. 21:121 (1951) (non Monanthotaxis angustifolia (Exell) Verdc., Kew Bull. 25: 21, 1971).
Popowia klainei Engl. var. angustifolia (Boutique) Le Thomas, Adansonia, sér. 2, 3: 291 (1963).
Monanthotaxis klainei (Engl.) Verdc. var. angustifolia (Boutique) Verdc., Kew Bull. 25: 21 (1971).

#### Note.

This species was described by Boutique (1951) based on the different leaf shape compared to *Monanthotaxis klainei* (Engl.) Verdc. The lack of other distinguishing features with *Monanthotaxis klainei* was the reason of Le Thomas (1963) and Verdcourt (1971) to treat it as a variety of *Monanthotaxis klainei*. However, they had only the availability of one flowering specimen. Now with an increased number of specimens available including fruiting material, we warrant it necessary to appraise this taxon again at species level. This species differs from *Monanthotaxis klainei* as mentioned by Boutique in the leaf shape, i.e. oblong to elliptic vs obovate in *Monanthotaxis klainei*, the rounded leaf base vs subcordate, the generally lower number of secondary veins (6–8 vs 7–12 in *Monanthotaxis klainei*) and it differs in the number of ovules per carpel (up to 6 vs 1 or 2 in *Monanthotaxis klainei*) and therefore also in the number of seeds per fruit. This species occurs sympatrically with Monanthotaxis klainei var. klainei, but differs from that species in having shorter pedicels (up to 12 mm in fruiting material vs 15–24 mm in fruiting material of Monanthotaxis klainei var. klainei). Actually, this species is much more similar in general aspects with *Monanthotaxis aquila* P.H.Hoekstra and *Monanthotaxis couvreurii* P.H.Hoekstra which are newly described in this article. It can be distinguished from those two species by the characteristics mentioned in Table 1. The epithet “atopostema” refers to the genus *Atopostema* Boutique in which it first was described. It is used as a noun.

**Table 1.** Differences between *Monanthotaxis aquila*, *Monanthotaxis couvreurii* and *Monanthotaxis atopostema*.

	*Monanthotaxis aquila*	*Monanthotaxis couvreurii*	*Monanthotaxis atopostema*
Nr of ovules per carpel	1–2	4	up to 6
Nr of staminodes	6	0	6?
Nr of stamens	9	13–15	9
Stamens free or basally connate	free	connate	free
Nr of carpels	12–13	9–12	ca 20
Nr of secondary veins	6–8	7–11	6–8
Distribution	Ivory Coast	Cameroon	Congo (Kinshasa)

### Monanthotaxis aquila

Taxon classificationPlantaeMagnolialesAnnonaceae

P.H.Hoekstra sp. nov.

urn:lsid:ipni.org:names:60472946-2

**Figure 1.**
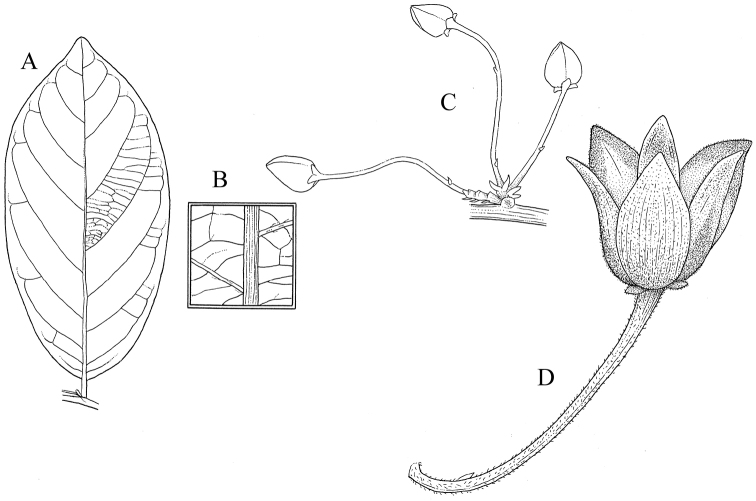
*Monanthotaxis aquila* P.H.Hoekstra. **A–H** drawn from the type (Geerling & Bokdam 2327). **A** Leaf **B** Leaf detail **C** Inflorescence **D** Flower. Illustration by M. Spitteler, reproduced from Hawthorne and Jongkind (2006).

**Figure 2.**
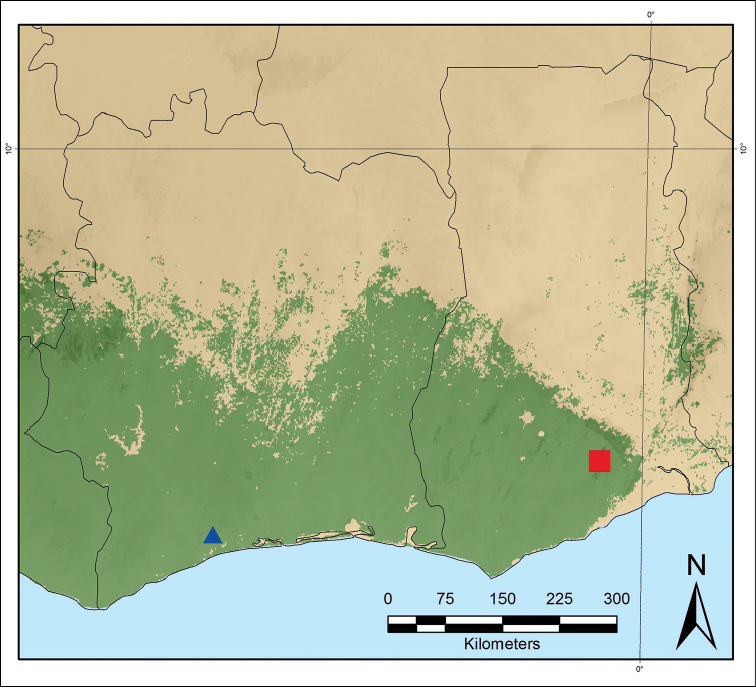
Distribution map of *Monanthotaxis aquila* (blue triangle) and *Monanthotaxis atewensis* (red square).

Figs 1 , 2 , Table 1

#### Type.

IVORY COAST. Sassandra, Dakpadou-Sago, 29 March 1968, *C. Geerling 2327* (holotype: WAG [2 sheets, barcodes: WAG0005568!, WAG0005569!]; isotype: BR [BR0000015315335!]).

#### Diagnosis.

Vegetatively similar to *Monanthotaxis atopostema* P.H.Hoekstra from Congo and *Monanthotaxis couvreurii* P.H.Hoekstra from Cameroon. Differs from the latter species in having staminodes alternating with the stamens, and free stamens. Differs from *Monanthotaxis atopostema* in having only 2 to 3 ovules per carpel, while the latter taxon has up to 6 seeds per monocarp.

#### Description.

*Liana*; young branches reddish brown with very short reddish brown hairs 0.1 mm, old branches dark brown, cylindrical with a few lenticels, glabrescent. **Leaves**: *petioles* 4–6 × 0.7–1.1 mm, not grooved, pubescence as branches; *lamina* 3.5–11.5 × 1.9–4.8 cm, length:width ratio 1.8–2.7, oblong, elliptic to slightly obovate, base cuneate to rounded, apex acute to acuminate, acumen to 1 cm, chartaceous, adaxially glossy dark green, abaxially dark silvery green, adaxially glabrous or midrib with a few short hairs, abaxially glabrescent, midrib of young leaves with short appressed yellowish hairs, venation eucamptodromous, secondary veins 6–8, from base curving upwards, tertiary venation scalariform. **Inflorescences** ramiflorous, axillary or supra-axillary, composed of a solitary flower or a 2 to 3 flowered rhipidium; sympodial rachis 0.5–2 mm with dense appressed short hairs; *flowering pedicels* 10–18 × 0.3–0.4 mm, with scattered appressed hairs; *lower bracts* lanceolate to ovate, 0.6–0.9 × 0.3–0.5 mm, indumentum as sympodial rachis; *upper bracts* at lower half of pedicel, ovate to lanceolate, 0.4–0.7 × 0.3–0.4 mm; *flower buds* ovate to triangular. **Flowers** bisexual; *sepals* 3, connate at base, 0.6 × 1.2–1.3 mm, broadly ovate, apex obtuse, with appressed yellowish hairs; *receptacle* flat, 1.6–2.0 mm in diameter; *petals* yellow, in two whorls of 3, but base of inner petals visible in bud; *outer petals*, 3.4–4.5 × 2.1–2.8 mm, ovate, outside with scattered yellowish appressed hairs, inside with very short appressed hairs at apex; *inner petals* 2.3–3.5 × 1.3–1.5 elliptic to slightly rhombic, outside with very short yellowish hairs at the apex and in the centre, inside only with hairs at the apex; *stamens* 9 in one whorl, free, clavate 0.7–0.8 mm, filaments 0.3–0.4 mm, anther cells lateral, connective slightly papillose, truncate, square from above, staminodes 6 alternating between the stamens, but wantingwhere in front of inner petals 0.3 mm, glabrous; *carpels* 12–13, 0.9–1.0 × 0.4–0.5 mm, ellipsoid, densely hairy, with 2–3 ovules, lateral, stigma subsessile, globose 0.1 mm, glabrous. **Fruits**: Unknown.

#### Distribution.

Ivory Coast, province Sassandra. Figure 2.

#### Ecology.

Secondary forest, on sandy soil.

#### Phenology.

Flowering the end of March.

#### Conservation status.

Proposed IUCN Red List Category: **Critically Endangered**
(CR): B2ab(iii), only known from the type collection and the forests of that area are under serious threat (Chatelain et al. 1996).

#### Etymology.

Aquila is the Latin word for eagle. This species is named after my son Arend, the Dutch word for eagle. Aquila is used here as a noun.

#### Discussion.

This species belongs to a group of species with bisexual flowers, ovate flower buds and predominantly cauliflorous or ramiflorous inflorescences. Most species of this group have obovate to oblanceolate leaves, whereas this species has oblong to elliptic-oblong leaves, a characteristic shared with *Monanthotaxis couvreurii* and *Monanthotaxis atopostema*. For the differences between those species see Table 1. *Monanthotaxis vogelii* (Hook. f.) Verdc. is similar to *Monanthotaxis aquila*, but differs in having obovate to oblanceolate leaves with the secondary veins being straight and only slightly curving upwards near the leaf margin and the petals are shorter. Vegetatively *Monanthotaxis mannii* (Baill.) Verdc. looks similar to *Monanthotaxis aquila*, but differs in having the inflorescences on the leafy twigs (vs rami- or cauliflorous), and rounded flower buds (vs ovate).

### Monanthotaxis atewensis

Taxon classificationPlantaeMagnolialesAnnonaceae

P.H.Hoekstra sp. nov.

urn:lsid:ipni.org:names:60472947-2

Figs 2 , 3

#### Type.

GHANA. Eastern Region, Atewa Range Forest Reserve, 2 June 1973, *J.B. Hall GC43672* (holotype: WAG [2 sheets, barcode WAG0019665!, WAG0019666!]; isotypes: GC!, K [K000040198!], MO [2189255]!)

*Monanthotaxis atewae* Hawthorne & Jongkind (2006: 72); invalid: description in English, no type designated (this termination, although admissible, does not follow recommandation 60D of the ICBN).

#### Diagnosis.

Easily distinguishable from all other *Monanthotaxis* species by the lanceolate sepals 1 cm long. Resembles *Monanthotaxis stenosepala* (Engl. & Diels) Verdc., but differs in the longer sepals, erect hairs on the leaves and branches, and a larger number of seeds per monocarp.

#### Description.

*Scandent shrub* or liana, to 9 m tall; young branches dark brown, with scattered erect reddish brown hairs 0.3–0.5 mm, old branches light brown, glabrescent with a few lenticels. **Leaves**: *petioles* 3–5 × 1.0–1.2 mm, grooved adaxially, indumentum as branches; *lamina* 5.7–15.9 × 2.4–5.4 cm, length:width ratio 2.0–3.1, oblong to slightly oblanceolate or obovate, base rounded, truncate or subcordate, apex acute to acuminate, acumen to 1.5 cm, chartaceous, abaxially greyish, adaxially glabrous, the midrib impressed with a few short hairs near the base, abaxially sparsely to densely covered with short erect reddish brown hairs, venation eucamptodromous, secondary veins 8–14, first straight halfway curving upwards, tertiary venation scalariform. **Inflorescences** leaf-opposed, composed of a solitary flower to a 3-flowered rhipidium; sympodial rachis 2–4 mm; *flowering pedicels* 21 × 0.7 mm, with short yellowish ascending to erect hairs; *lower bract* strongly reduced or wanting; *upper bract* around the middle of the pedicel, ovate, 1.7 × 1.4 mm, densely covered with short appressed hairs; *sepals* 3, free, 10–12 × 2.6–2.7 mm, lanceolate, apex acute, with dense short appressed hairs; *receptacle* 3.0 mm in diameter, flat; *petals* unknown; *stamens* unknown, scars in a single whorl; *carpels* 1.2 × 0.5 mm, ellipsoid, dense hairy, stigma elongate 0.5 mm, grooved, glabrous. **Fruits**: *pedicels* 20–37 × 0.6–1.1 mm; *sepals* persistent or caducous; *stipes* 4–6 mm long, slightly grooved; *monocarps* 1–9, ellipsoid with 1 to 5 seeds, 13–35 × 5–6 mm, slightly to strongly constricted between the seeds, slightly verrucose, apex apiculate, apex to 2.0 mm, with scattered short erect hairs, unripe fruits green**. Seeds** 9 × 6 mm, ellipsoid, base and apex rounded, tawny brown, raphe visible from base to apex, ruminations lamelliform.

**Figure 3.**
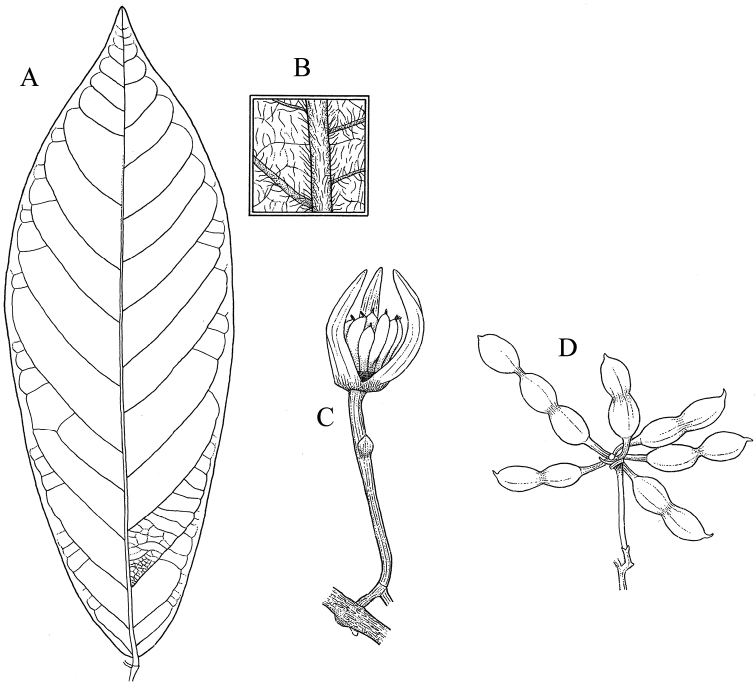
*Monanthotaxis atewensis* P.H.Hoekstra. **A, B, D** drawn from the type (Hall & Lock GC43672) **C** drawn from Hall & Enti GC36426. **A** Leaf **B** Leaf detail **C** Old flower **D** Fruit. Illustration by M. Spitteler, from Hawthorne and Jongkind (2006).

#### Distribution.

Ghana, Eastern Region, Atewa Range Forest Reserve. Figure 2.

#### Ecology.

Forest, in thicket, at 750 m altitude.

#### Phenology.

Fruiting in May and June.

#### Conservation status.

Proposed IUCN Red List Category: **Critically Endangered**
(CR): B2ab(iii), only known from the Atewa Range Forest Reserve and although it is a protected area, the forest is under threat of bauxite mining and logging (Kusimi 2015; Ntiamoa-Baidu et al. 2000). Furthermore, the species has not been collected in more than 40 years.

#### Etymology.

Named after the Atewa Range Forest Reserve in Ghana, to which this species seems to be endemic.

#### Additional specimens examined (paratypes).

**GHANA. Eastern Region**: Atewa Range Forest Reserve, 12 May 1967, *J.B. Hall GC36426* (K [K000040199], WAG [WAG0019664]).

#### Discussion.

This species can easily be distinguished from all other species of *Monanthotaxis* by the large lanceolate sepals. The species is similar to *Monanthotaxis stenosepala* (Engl. & Diels) Verdc., which also has lanceolate sepals and light brown older branches. However, the sepals of *Monanthotaxis stenosepala* are 4 to 6 mm vs 10 to 12 mm in *Monanthotaxis atewensis* and the pubescence and number of seeds is different as described in the diagnosis. Two fruiting specimens from Liberia (*Stoop 331* and *Adam 26189*) closely resemble *Monanthotaxis atewensis*, but no sepals are present to verify the identification. Furthermore, the monocarps are more densely verrucose and the peduncle is shorter and the pedicel larger than the two specimens of *Monanthotaxis atewensis* from Ghana. More material or more recent material for DNA extraction is needed to assess the status of those specimens from Liberia.

For now we consider this species an endemic to the Atewa Range. This is the first plant species that is endemic to this mountain range. However, for several Upper Guinean endemics (e.g. *Dorstenia embergeri* Mangenot) this range is their most eastern outpost. Some other plants are only known from these mountains and one or two other localities in Ghana. The Atewa Range is home of 3 endemic butterfly species (McCullough et al. 2007). This new endemic urges for a strict protection of this unique mountain chain.

### Monanthotaxis couvreurii

Taxon classificationPlantaeMagnolialesAnnonaceae

P.H.Hoekstra sp. nov.

urn:lsid:ipni.org:names:60472948-2

**Figure 4.**
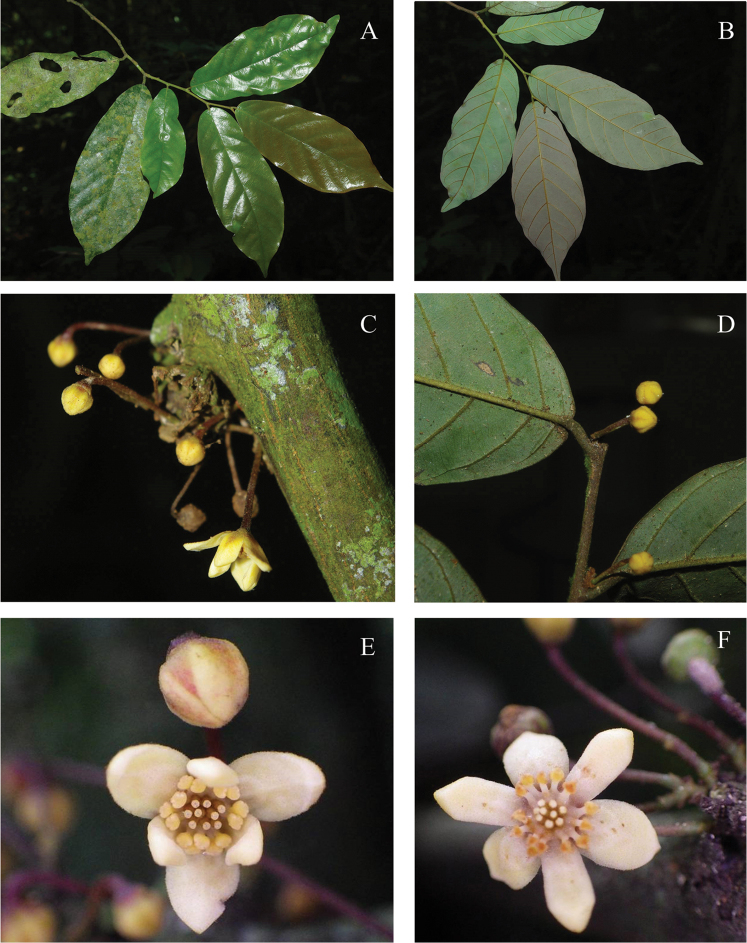
*Monanthotaxis couvreurii* P.H.Hoekstra. **A–F** photographs in the field of the type collection (TLP Couvreur 762). Photos: Thomas Couvreur.

**Figure 5.**
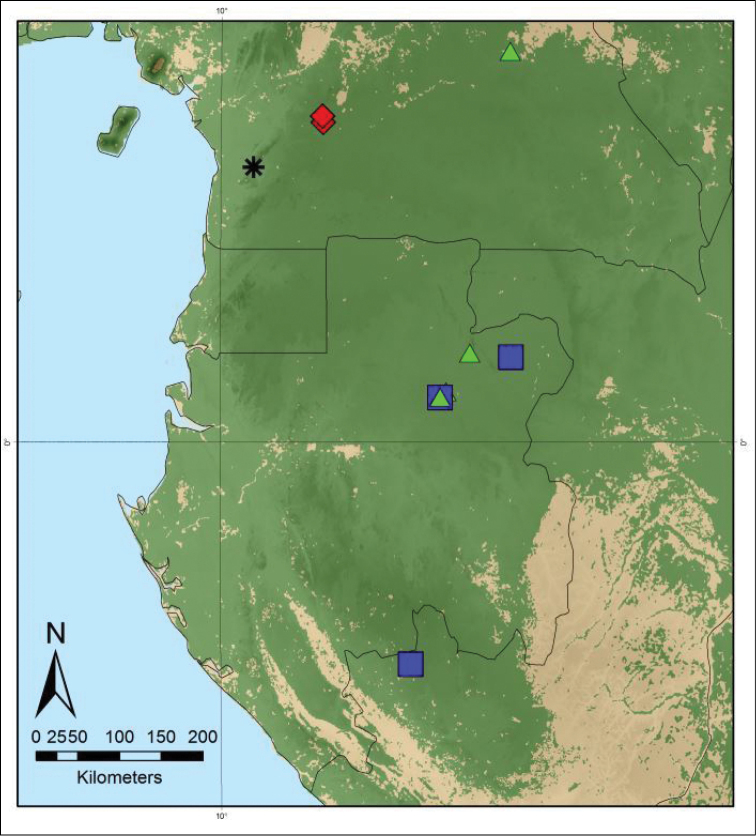
Distribution map of *Monanthotaxis couvreurii* (red diamonds), *Monanthotaxis latistamina* (blue squares), *Monanthotaxis tripetala* (green triangles) and *Monanthotaxis zenkeri* (black asterisk).

Figs 4 , 5 , Table 1

#### Type.

CAMEROON, Central Province, Ottotomo Forest Reserve, 45 km SW of Yaoundé, ca 5 km on main path into reserve. 3°35.21'N; 11°17.63'E, 24 April 2015, *T.L.P. Couvreur* 762 (holotype: WAG [3 sheets, barcodes: WAG.1576998!, WAG.1576999!, WAG.1577000!]).

#### Diagnosis.

Differs from all *Monanthotaxis* species by the stamens that are basally connate. The leaves are similar to *Monanthotaxis atopostema* P.H.Hoekstra from Congo and *Monanthotaxis aquila* P.H.Hoekstra from Ivory Coast, but the flowers of *Monanthotaxis couvreurii* differ in having no staminodes and the leaves have more secondary veins with smaller spaces in between.

#### Description.

*Liana*; young branches reddish brown with dense ascending reddish brown hairs 0.1–0.2 mm, old branches greyish brown, slightly grooved,. **Leaves**: *petioles* 3–5 × 0.8–0.9 mm, slightly grooved, indumentum as branches; *lamina* 4.5–12.0 × 1.8–4.3 cm, length:width ratio 2.1–2.9, oblong to obovate, base cuneate to rounded, apex acute to acuminate, acumen to 1 cm, chartaceous, discolorous, adaxially glossy green, abaxially light greyish green, adaxially sparsely covered with whitish appressed hairs 0.1 mm, soon glabrescent, abaxially with scattered appressed whitish yellowish hairs 0.1–0.2 mm, , venation eucamptodromous, secondary veins 7–11, from base curving upwards, tertiary venation scalariform sometimes obscure. **Inflorescences** cauliflorous, ramiflorous or axillary, composed from a two-flowered rhipidium in the axils of the leaves to many-flowered clusters on the trunk; sympodial rachis 1–15 mm; *flowering pedicels* 4–20 × 0.2–0.6 mm, with scattered ascending to erect hairs 0.1 mm; *lower bracts* strongly reduced or wanting; upper bracts wanting; *flower buds* ovate. **Flowers** bisexual; *sepals* 3, 0.8–0.9 × 0.9–1.0 mm, triangular, apex acute, with dense yellowish hairs; *receptacle* flat, 1.2–2.0 mm in diameter; *petals* light yellow to white, in two whorls of 3, but base of inner petals visible in bud; *outer petals*, 3.5–5.0 × 2.0–3.5 mm, elliptic-ovate, outside with dense short yellowish hairs, inside with a few hairs near the margins; *inner petals* 3.0–4.5 × 1.2–1.5 mm, elliptic to narrowly ovate, outside with yellowish hairs at the apex and at the centre, inside glabrous or with a few hairs at the margins; *stamens* 13–15 in one whorl, connate at base, linear-obconic 0.8–0.9 mm, filaments 0.4 mm, anther cells lateral to extrorse, connective papillose, truncate, rounded from above, staminodes 0; *carpels* 9–12, 1.2–1.3 × 0.3–0.4 mm, subcylindric to ellipsoid, dense hairy, with 4 lateral ovules, stigma subsessile 0.2 mm, globose, glabrous. **Fruits**: Not seen, but according to collection Farron 7359 with 4 articles.

#### Distribution.

Cameroon, Central Province, Ottotomo Forest Reserve. Figure 5.

#### Ecology.

Old secondary forest, on slope, at 700 m altitude.

#### Phenology.

Flowers collected in April and May.

#### Conservation status.

Proposed IUCN Red List Category: **Critically Endangered**
(CR): B2ab(iii), only known from the Ottotomo Forest Reserve in Cameroon. The increase of human population around the reserve intensifies the pressure on the forest, while the surrounding forests are increasingly degrading (Sassen and Jum 2007), warranting the critically endangered status of this species.

#### Etymology.

Named after Thomas L.P. Couvreur, a passionate Annonaceae systematist and collector of the type of this species and of *Monanthotaxis latistamina* P.H.Hoekstra also described in this article.

#### Additional specimens examined (all paratypes).

**CAMEROON. Central Province**: Ottotomo reserve, 5 May 1970, *C. Farron 7266* (P [P01954685]); Ottotomo reserve, 26 May 1970, *C. Farron 7359* (P [P01954686], YA [YA0044284]).

#### Discussion.

This species belongs to a group of species with predominantly ramiflorous inflorescences, bisexual flowers and ovate flower buds and looks vegetatively very similar to *Monanthotaxis aquila* and *Monanthotaxis atopostema*. For the differences between these species see Table 1. *Monanthotaxis couvreurii* can be distinguished from all *Monanthotaxis* species in having the stamens basally connate (see figure 4F). *Monanthotaxis klainei* (Engl.) Verdc. also has the stamens connate in one whorl, but in that species the stamens are alternating with staminodes.

### Monanthotaxis filipes

Taxon classificationPlantaeMagnolialesAnnonaceae

P.H.Hoekstra sp. nov.

urn:lsid:ipni.org:names:60472949-2

**Figure 6.**
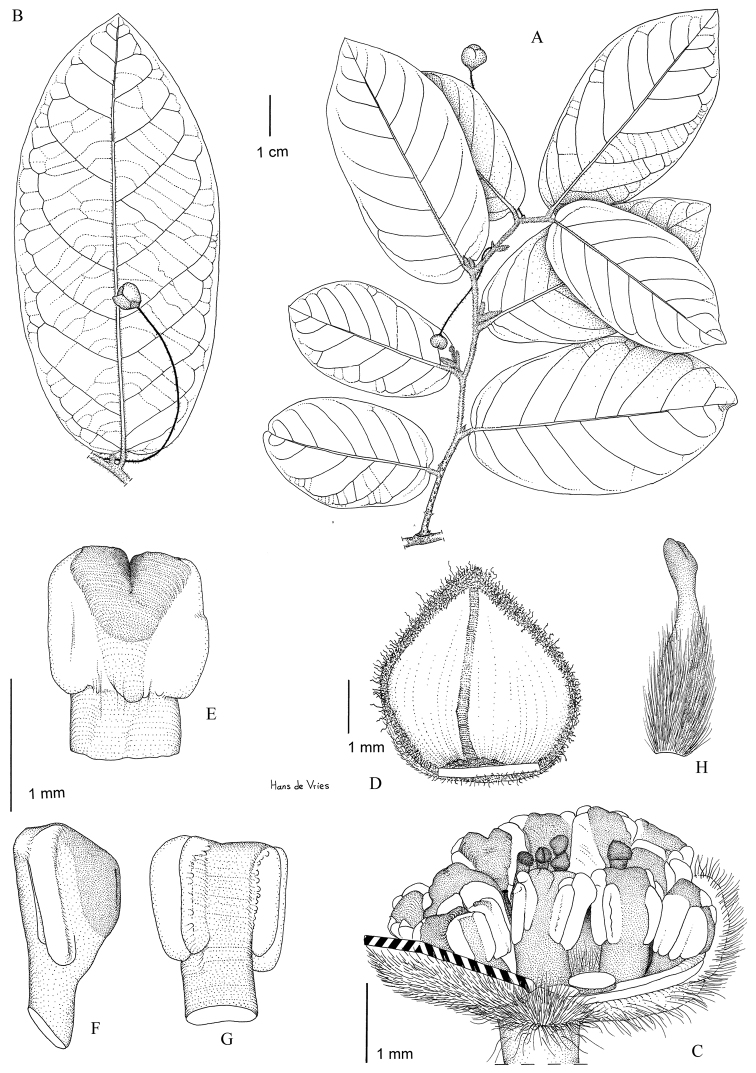
*Monanthotaxis filipes* P.H.Hoekstra. **A–H** drawn from the type (Bidgood 1402). **A** Habitus **B** Leaf with flower **C** Flower with petals removed **D** Outer petal inside **E** Stamen inside **F** Stamen lateral **G** Stamen outside **H** Carpel. Illustration by H. de Vries.

**Figure 7.**
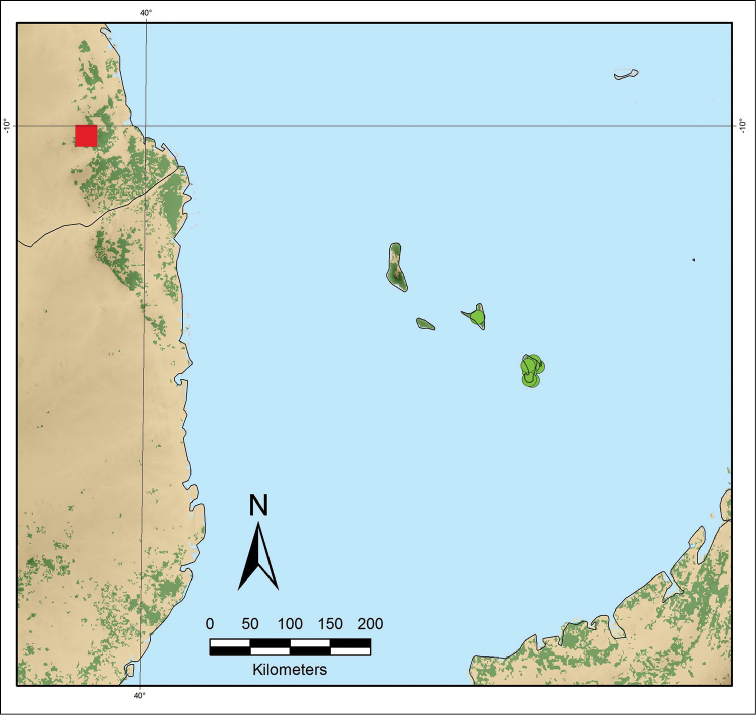
Distribution map of *Monanthotaxis filipes* (red square) in Tanzania and *Monanthotaxis komorensis* (green circles) on the Comoros archipelago.

Figs 6 , 7 , Table 2

#### Type.

TANZANIA, Lindi district, Rondo plateau, Rondo forest Reserve, 10°07'S; 39°13'E, 7 February 1991, *G.S. Bidgood 1402* (holotype: K [2sheets!]; isotypes: BR [BR0000013186036!], C!, EA!, MO [4027188!], P [P01967237!], UPS!, WAG [WAG0071696!]).

#### Diagnosis.

This species is similar to *Monanthotaxis trichantha* (Diels) Verdc. because of the dense yellow short indumentum on the young stems. It differs in the pendulous flowers on filiform pedicels, and in the outer petals covering the inner petals in bud.

#### Description.

*Shrub*, 1 m tall; young branches yellow-brown, with dense appressed to ascending yellowish hairs 0.3–0.7 mm, old branches dark brown to greyish brown, cylindrical, glabrescent with many lenticels. **Leaves**: *petioles* 3–5 × 0.7–1.0 mm, indumentum as branches; *lamina* 3.7–10.7 × 1.5–4.4 cm, length:width ratio 1.6–3.3, oblong to elliptic to slightly obovate, base rounded, truncate or slightly subcordate, with thickened margin at base, apex acute, chartaceous, discolorous, abaxially pale bluish green adaxialy soon glabrescent, with whitish appressed hairs when young, abaxially densely covered with ascending yellowish white hairs, venation eucamptodromous, midrib impressed adaxially, secondary veins 7–10, first straight halfway curving upwards, tertiary venation scalariform. **Inflorescences** supra-axillary, 2–6 mm above leaf axil, 1–2-flowered rhipidium; sympodial rachis 0–1 mm; *flowering pedicels* filiform 18–55 × 0.2 mm, with a few scattered ascending to erect hairs; *lower bract* strongly reduced or wanting; *upper bract* halfway the pedicel, very small, 0.3 × 0.1 mm or just a dense tuft of hairs or wanting; *flower buds* rounded. **Flowers** bisexual, pendulous; *sepals* 3, connate at the base, broadly ovate to almost cupuliform, 0.5 × 1.1–1.2 mm, obtuse to rounded, with dense yellow appressed hairs; *receptacle* 1.5–2 mm in diameter, flat; *petals* creamy white, 6 in two whorls; *outer petals*, 2.5–2.7 × 2.8–3.6 mm, broadly ovate, densely covered with short yellowish hairs on the outside and apex of the inside, glabrous at base of inside; *inner petals* 1.9–2.0 × 1.2–1.4 mm, ovate to rhombic, indumentum as in outer petals; *stamens* (13?–)15 in one to two whorls, free, flattened obovate, 1.1–1.3 mm long, filaments 0.3–0.4 mm long, anthers extrorse, connective truncate, not hiding anther cells, glabrous, staminodes 0; *carpels* 9, 1.2 × 0.2–0.3 mm, subcylindric, densely hairy, but glabrous near apex, with 2 lateral ovules, stigma elongate 0.4 mm, grooved, glabrous. **Fruits** unknown.

#### Distribution.

Tanzania, Lindi Region. Figure 7.

#### Ecology.

Steep escarpment with dense thicket, stony-gravelly soil at 700 m altitude.

#### Phenology.

Flowers collected the 7^th^ of February.

#### Conservation status.

Proposed IUCN Red List Category: **Critically Endangered**
(CR): B2ab(ii, iii), only known from one collection in the Rondo Forest Reserve in South-east Tanzania. Although it occurs in a forest reserve, satellite images provided by Google Earth (assessed May 2016) show that a major part of the forest in the reserve systematically has been removed, and forest cover is declining over the years.

#### Etymology.

From the latin *fili*, thread-like, and *pes*, foot/stalk; referring to the filiform pedicels.

#### Discussion.

This species is vegetatively very similar to *Monanthotaxis trichantha* which also occurs in South-East Tanzania. The flowers and inflorescences are very different with *Monanthotaxis filipes* having pending flowers on long pedicels and the inflorescences supra-axillary, while *Monanthotaxis trichantha* has short pedicels which are extra-axillary or leaf-opposed (table 2). Apart from *Monanthotaxis filipes*, long filiform pedicels only occur in some species of *Monanthotaxis* from Madagascar (e.g. *Monanthotaxis caesia* (Diels) Verdc. and *Monanthotaxis heterantha* (Baill.) Verdc. These species lack the dense indumentum on the young twigs. Also *Monanthotaxis oligandra* Exell has pending supra-axillary inflorescences, but in this species the sympodial rachis (peduncle) is filiform instead of the pedicels, which are very short. Furthermore, *Monanthotaxis oligandra* lacks the dense yellow pubescence and has very different flowers with all petals in a single whorl.

**Table 2.** Differences between *Monanthotaxis filipes* and *Monanthotaxis trichantha*.

	*Monanthotaxis filipes*	*Monanthotaxis trichantha*
Flowering pedicel length	18–55 mm	2–8 mm
Inflorescence position	supra-axillary	extra-axillary/leaf opposed
Number of petals visible in bud	3	6
Outer petals form	broadly ovate	ovate to elliptic
Inner petals form	ovate	elliptic

### Monanthotaxis komorensis

Taxon classificationPlantaeMagnolialesAnnonaceae

P.H.Hoekstra sp. nov.

urn:lsid:ipni.org:names:60472950-2

Figs 7 , 8

#### Type.

MAYOTTE, Grande Terre, Mont Combani, départ du GR menant au sommet, 10 January 2002, *F. Barthelat 671* (holotype: P [P00273165!]; isotypes: G [G00404210!], K, MAYOT, MO [5735265!]).

#### Diagnosis.

Differs from all other *Monanthotaxis* species in the combination of having solitary flowers on a short pedicel with 6 staminodes alternating with 6 stamens.

#### Description.

*Liana* or shrub to 2m; young branches dark reddish brown with ascending to slightly erect yellowish hairs 0.2 mm, old branches greyish brown to blackish brown, glabrescent with lenticels. **Leaves**: *petioles* 2–3(–4) × 1.4–1.6 mm, grooved adaxially, slightly more dense indumentum as branches; *lamina* 4.0–17.0 × 1.8–5.7 cm, length:width ratio 2.2–3.4, oblong to elliptic lanceolate, base subcordate, apex acute, chartaceous, discolorous, adaxially glossy green, abaxially glaucous, adaxially glabrous or with a few yellowish hairs at the base of the midrib, abaxially with scattered short ascending whitish yellowish hairs 0.1–0.3 mm, soon glabrescent, venation festooned brochidodromous, secondary veins (8–)13–18, from base curving upwards, tertiary venation reticulate, adaxially slightly raised. **Inflorescences** axillary, leaf-opposed or terminal, composed of a solitary flower; sympodial rachis reduced or wanting; *flowering pedicels* 3–6 × 0.4–0.5 mm with ascending to erect hairs 0.1–0.2 mm; *lower bracts* wanting; *upper bracts* halfway the pedicel, ovate to lanceolate, 0.3–0.8 × 0.4–0.5 mm; *flower buds* rounded to slightly ovate. **Flowers** bisexual; *sepals* 3, 0.6–0.8 × 1.4–1.6 mm, broadly ovate, with short yellowish hairs, apex obtuse; *receptacle* flat, 1.8–2.5 mm in diameter; *petals* 6 in two whorls, creamy yellow with pinkish to reddish brown base of the inner petals; *outer petals* 5.5–7.0 × 5.3–5.4 mm, ovate, outside with short yellowish appressed hairs, inside with very short hairs, but glabrous at base; *inner petals* 4.2–4.4 × 3.0–3.3 mm, rhombic, outside with short yellowish hairs, inside with very short hairs, but glabrous at base; *stamens* 6–7 in one whorl, free, obconic 1.2 mm, filaments 0.4 mm, anthers lateral to introrse, connective glabrous, truncate, prolonged inward, staminodes 6 alternating between the stamens, 0.7 mm, glabrous; *carpels* 8, 1.4–1.5 × 0.2 mm, subcylindric, densely hairy, with 2 lateral ovules, stigma elongate 0.6 mm, with a few hairs at base. **Fruits**: *pedicels* 9 × 0.9 mm; *sepals* persistent, slightly acrescent; *stipes* 2.0–2.5 mm; *monocarps* up to 7, ellipsoid with 1 or 2 seeds, 10–17 × 5–6 mm, slightly constricted between the seeds, apex rounded, glabrous or with a few short hairs, verrucose, ripe fruits red. **Seeds** 6 × 5 mm, ellipsoid, apex slightly apiculate, flattened were touching with other seed, ochre-brown, raphe slightly visible as a longitudinal furrow from base to apex, ruminations lamelliform.

**Figure 8.**
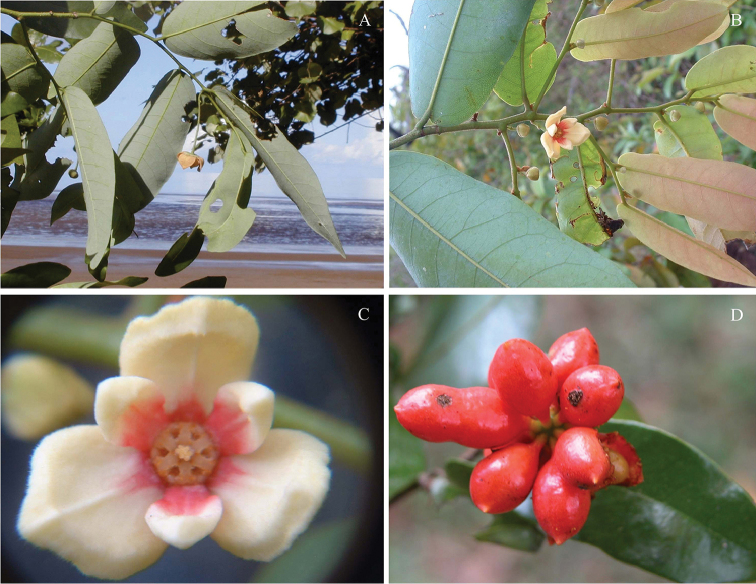
*Monanthotaxis komorensis* P.H.Hoekstra. **A** Photographs in the field of collection Barthelat 833 **B–C** idem, Barthelat 1045, **D** idem, Barthelat 1319. Photos: Fabien Barthelat.

#### Distribution.

Mayotte, Grande Terre and Mbouzi; Comoros, Anjouan. Figure 7.

#### Ecology.

Not rare in humid and lowland hill forests, including secondary vegetation. It is also found in shady places in dry forest, especially along riversides.

#### Phenology.

Flowering from November to January, fruits collected in January and April.

#### Vernacular names.

Shibushi language: Fotsy ambadiki (*F. Barthelat 590*), Fotsy Ambadiky bé (*F. Barthelat 1045*), Fotsy ambadiky lahy (*F. Barthelat 1045*), Foutsi Ambadiki (*O. Pascal 280*), Sari langilangy (*H. Ralimanana 277*), Tchavadiki (*O. Pascal 338*).

#### Conservation status.

Proposed IUCN Red List Category: **Vulnerable**
(VU): B2ab(I, ii, iii, iv), EOO (including Anjouan) 1231 km^2^, AOO 32 km^2^, 9 locations. This species has probably disappeared from the island Anjouan as the only collections were made more than 100 year ago, however there are 9 recent collections from Mayotte from different localities on the island Grande Terre of which some in nature reserves and one on Mbouzi, a protected island. The vegetation of the island Mayotte remains under threat, likely reducing the population of *Monanthotaxis komorensis* in the future.

#### Etymology.

Named after the Comoro Archipelago. Komorensis is written with a K as in the Latin script of the Comorian language and Shimaore.

#### Additional specimens examined (all paratypes).

**COMOROS. Anjouan**: *Lavanchie 24* (P [P00213916]) and *Lavanchie 25* (P [P00213917]). **MAYOTTE. Grande Terre**: Mamoudzou, reserve forestière de Majimbini, 24 Jan 2001, *F. Barthelat 269* (G [G00404208], K, MAYOT, MO, P [P00229255]); Mont Combani, 15 Nov 2001, *F. Barthelat 590* (G [G00404209], K, MAYOT, MO [5730093], P [P00273086]); Tsararano, Tchaourembo, 17 Oct 2002, *F. Barthelat 1045* (MAYOT, P [P00290506]); Mlima Combani, 28 Dec 1995, *O. Pascal 280* (P [P00127022]); Dapani, 17 Jan 1996, *O. Pascal 338* (P [P00127021]); Mlima Combani, 15 April 1996, *O. Pascal 487* (G [G00404211], K, MO, P [P00127020]); Mont Combani, 12°57.44'S, 45°07.75'E, 16 Nov 2002, *H. Ralimanana 277* (G [G00404207], K, MAYOT, P [P00538263], TAN); vallon à station pompage Ouroveni, 8 Nov 1989, *H. Tinguy 1028* (P [P01987602]). **Ilot MBouzi**: 12°48.33'S, 45°14.00'E, 26 April 1999, *M.M. Pignal 1285* (P [P00176736]).

#### Discussion.

This is the only known species of *Monanthotaxis* on the Comoros and Mayotte. It is easily distinguished from almost all other *Monanthotaxis* species by having 6 staminodes alternating with 6 stamens, all in a single whorl. The only other species sharing that feature, viz. *Monanthotaxis congoensis* Baillon and *Monanthotaxis paniculata* P.H.Hoekstra are very different. These have a single whorl of petals (vs two whorls in *Monanthotaxis komorensis*), inflorescences of raceme-like or panicle-like rhipidia, their young branches are densely covered with yellowish brown short hairs and they occur in Central Africa. The flowers of *Monanthotaxis glaucocarpa* (Baill.) Verdc., a species from Madagascar, are not known. This species differs from *Monanthotaxis komorensis* in having longer pedicels (>30 mm vs 9 mm), longer stipes (7–9 mm vs 2.0–2.5 mm) and longer and bigger seeds (12–15 mm vs 6 mm). Further, the leaves are less elongate and the young branches are glabrous.

### Monanthotaxis latistamina

Taxon classificationPlantaeMagnolialesAnnonaceae

P.H.Hoekstra sp. nov.

urn:lsid:ipni.org:names:60472951-2

**Figure 9.**
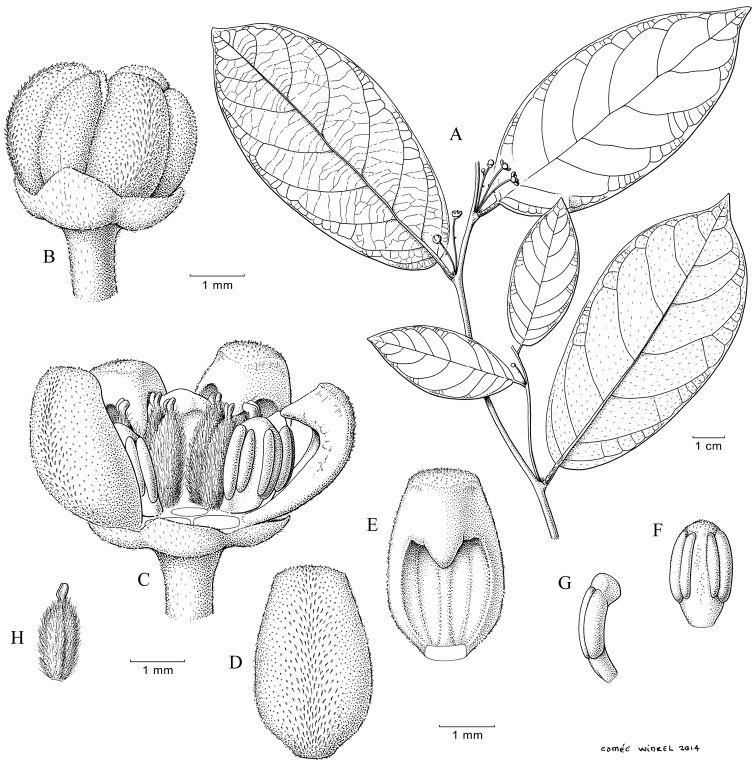
*Monanthotaxis latistamina* P.H.Hoekstra. A-H drawn from the type (Couvreur 565). **A** Habitus **B** Flower bud **C** Open flower with 2 petals and one stamen removed **D** Outer petal outside **E** Outer petal inside **F** Stamen inside **G** Stamen lateral **H** Carpel. Illustration by E. Winkel.

**Figure 10.**
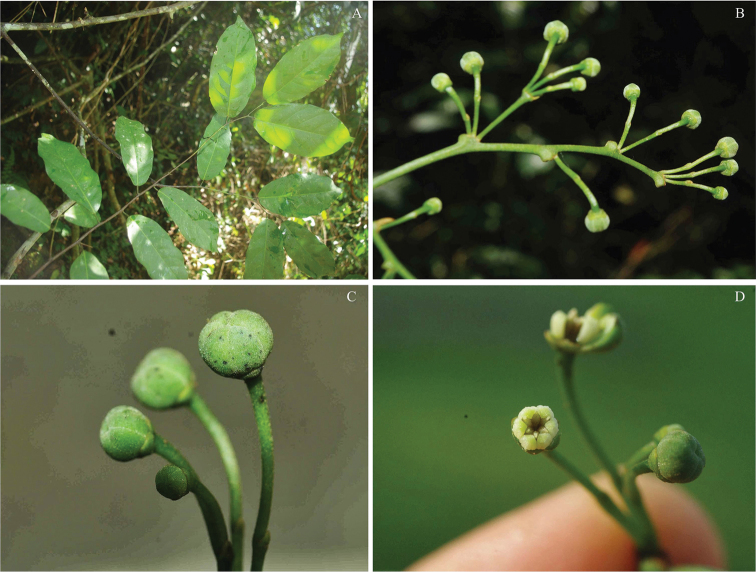
*Monanthotaxis latistamina* P.H.Hoekstra. **A–D** Photographs in the field from the type collection (TLP Couvreur 565). Photos: Thomas Couvreur.

Figs 5 , 9 , 10 , Table 3

#### Type.

GABON. Ogooué-Ivindo, Ivindo National Park, along main trail departing from behind the herbarium at the Research station of Ipassa, 0°30.23'N, 12°47.59'E, 11 November 2013, *T.L.P. Couvreur 565* (holotype: WAG [3 sheets, barcodes: WAG.1577028!, WAG.1577029!, WAG.1577030!]; isotype: LBV, YA).

#### Diagnosis.

Closely related to *Gilbertiella congolana* Boutique by the papillose petals and stamens. Differs from *Gilbertiella congolana* by having 6 oblong stamen (vs 12 linear stamen) which are wider than deep (vs rounded in cross-section).

#### Description.

*Scandent shrub* or liana to 4 m high; young branches sparsely pubescent with appressed yellowish hairs 0.1–0.2 mm, old branches dark, blackish, soon glabrescent sometimes with a few lenticels. **Leaves**: *Petioles* 5–12 × 1.3–1.7 mm, grooved adaxially, indumentum as branches; *lamina* 6–13 × 3.5–7.2 cm, length:width ratio 1.6–2.7, oblong or elliptic to slightly obovate, base cuneate, rounded to subcordate with thickened black margin at base, apex acute to acuminate, acumen to 1.3 cm long, chartaceous to subcoriaceous, adaxially green, abaxially light greyish green, young leaves with a few short appressed hairs, soon glabrescent, venation eucamptodromous, secondary veins 7–8(–10) oblique, from base curving upwards, tertiary venation reticulate, raised adaxially, leaves are punctate, although difficult to see in older leaves. **Inflorescences** axillary or terminal, solitary, in fascicle-like rhipidia or in a to 4 cm long short lax panicle-like rhipidium with up to 6 flowers; sympodial rachis appressed pubescent to glabrescent; *flowering pedicels* 8–17 × 0.3–0.4mm, indumentum as sympodial rachis; *lower bracts* strongly reduced or wanting; *upper bracts* lanceolate to ovate 0.6–0.7 × 0.4–0.6 mm, same pubescence as sympodial rachis, placed halfway the pedicel; *flower buds* globose to slightly ellipsoid. **Flowers** bisexual; *receptacle* 1.2–1.5 mm in diameter; *sepals* 3, united at base, 0.8–1.0 ×1.2–1.5 mm, ovate to broadly ovate, adaxially with short appressed yellowish hairs, apex obtuse to slightly acute; *petals* 6 in one whorl, but 3 petals overlapping others at apex in bud, in young flowers green, 1.6–2.5 × 0.7–1.2 mm elliptic to slightly ovate, with inwardly reflexed appendage at apex, outside papillose to slightly short appressed pubescent, inside papillose, most dense at apex; *stamens* 6, in one whorl, alternate with petals, light green, oblong, densely papillose wider than thick 1.0–1.2 × 0.6–0.7 mm, radial width 0.2–0.3 mm, filaments 0.1 mm, anthers 2, extrorse, connective appendage small, just visible between the anthers, densely papillose, truncate; *staminodes* 0; *carpels* 6–9, 1.0–1.4 × 0.4–0.6 mm, ellipsoid densely reddish brown pubescent with 6 lateral ovules, stigma globose to slightly elongated, grooved to almost bifurcate, 0.1–0.2 mm, glabrous. **Fruits** reported as yellowish, not seen.

#### Distribution.

Gabon, province Ogooué-Ivindo. Republic of Congo, province Niari. Figure 5.

#### Ecology.

Evergreen forest and forest on shallow soil at summit of hill, elev. 519–1017 m.

#### Phenology.

Flowers collected in May, October and November.

#### Conservation status.

Proposed IUCN Red List Category: **Endangered**
(ED): B2ab(iii), EOO 17682 km^2^, AOO 16 km^2^. Although the species has quite a wide distribution, it is only known from four collections, one of which is from a protected area. The other collections are from areas under threat of logging and habitat destruction, which is why we suggest the status of endangered.

#### Etymology.

Named latistamina for the wide, but flattened stamens, which differentiates it from the similar *Gilbertiella congolana* Boutique.

#### Additional specimens examined.

**REPUBLIC OF CONGO. Niari**: Edge of the Missanda plain, 28 October 1975, *P. Sita 3966* (P [P01982495],P [P01982496] , WAG [WAG.1575264]; excluded as paratype). **GABON. Ogooué-Ivindo**: Ca 4 km NNW of Ikei-Bokaboka, 0°57.18'N, 13°41.45'E, 18 May 2003, *L. Ngok Banak 1811* (paratypes: BRLU, LBV [LBV0001649], MO [6357151], P, WAG [WAG0148822]; Belinga mountain, 1°04.87'N; 13°12.18'E, 11 November 2015, *J.J. Wieringa 8320* (paratypes: WAG [3 sheets, WAG.1575172, WAG.1575173, WAG.1575174]).).

#### Discussion.

This species is similar to *Gilbertiella congolana* Boutique from Congo (Kinshasa), but it differs in stamen number and form, furthermore *Monanthotaxis latistamina* has thicker petioles, while the petioles of *Gilbertiella congolana* are more slender (Table 3).

**Table 3.** Differences between *Monanthotaxis latistamina* and *Gilbertiella congolana*.

	*Monanthotaxis latistamina*	*Gilbertiella congolana*
Nr of stamens	6	12–15
Stamen form	oblong, wider than thick	obconical to clavate, circular in cross-section
Petiole width	1.3–1.7 mm	0.7–0.8 mm

**Figure 11.**
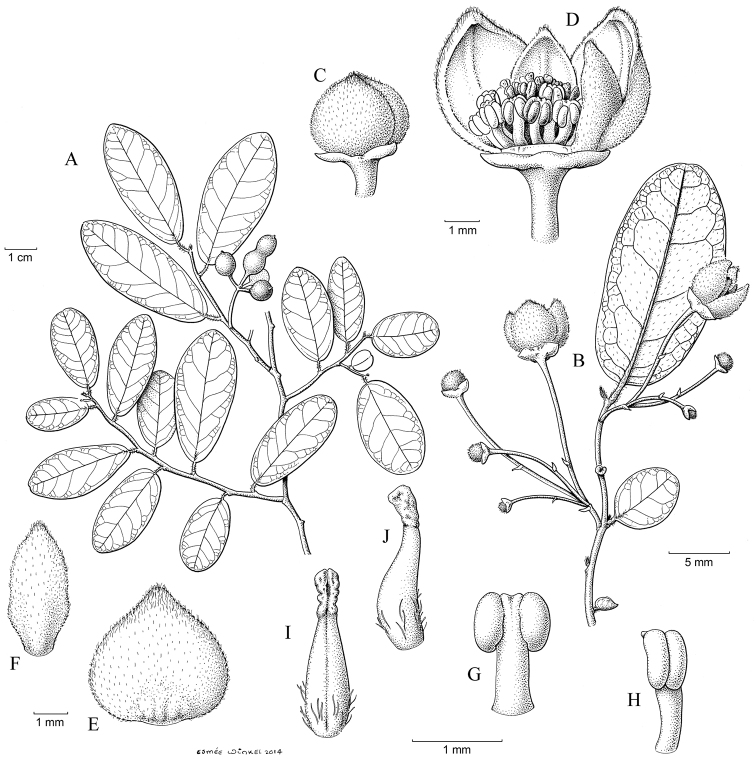
*Monanthotaxis maputensis* P.H.Hoekstra. **A** drawn from Mendonça 4480 **B–J** drawn from Exell 565 **A** Habitus with fruit **B** Habitus with flowers **C** Flower bud **D** Open flower with 2 petals removed **E** Outer petal **F** Inner petal **G** Stamen inside **H** Stamen lateral **I** Carpel inside **J** Carpel lateral. Illustration by E. Winkel.

Although *Monanthotaxis latistamina* is similar to *Gilbertiella congolana* we do describe it in *Monanthotaxis* as *Gilbertiella* Boutique will be synonymized with *Monanthotaxis*. Boutique (1951) described *Gilbertiella* based on linear stamens, outer petals that cover the inner petals in bud only at the apex, and an apical appendage on the inside of the petals. These characters on their own occur in other species of *Monanthotaxis* as well. The outer petals overlapping the inner petals only at the apex occurs in a quarter of all *Monanthotaxis* species, the linear stamens occur in some species such as *Monanthotaxis filamentosa* (Diels) Verdc. and *Monanthotaxis maputensis* P.H.Hoekstra (figure 11) and a more or less developed appendage on the petals occurs in species such as *Monanthotaxis le-testui* Pellegrin and male flowers of *Monanthotaxis cauliflora* (Chipp) Verdc. and often in young flower buds. All other characteristics of *Gilbertiella congolana* are typical or at least normal for *Monanthotaxis*, such as having only a few stamens in one whorl and uniseriate stipitate monocarps. Also Walker (1971) reported a strong affinity between *Monanthotaxis* and *Gilbertiella* based on the microbaculate pollen exine. Based on all these similarities we place *Monanthotaxis latistamina* in the genus *Monanthotaxis*. Furthermore, DNA sequences confirm the placement of *Monanthotaxis latistamina* within the genus *Monanthotaxis* (Guo et al. in press).

The specimen of *Monanthotaxis latistamina* from the Republic of Congo has some differences with the three specimens from Gabon. The flower buds and stems of the specimen dried very blackish and the leaves are coriaceous and greyish vs chartaceous and green in the Gabonese specimens. However the distinguishing characteristics with *Gilbertiella congolana* are the same as with the Gabonese specimens. More collections are needed to verify if this is just an aberrant collection or a different (sub)species. For now this collection is retained as belonging to *Monanthotaxis latistamina* based on the similarities in flowers and stamen, but it is excluded as a paratype.

### Monanthotaxis maputensis

Taxon classificationPlantaeMagnolialesAnnonaceae

P.H.Hoekstra sp. nov.

urn:lsid:ipni.org:names:60472952-2

**Figure 12.**
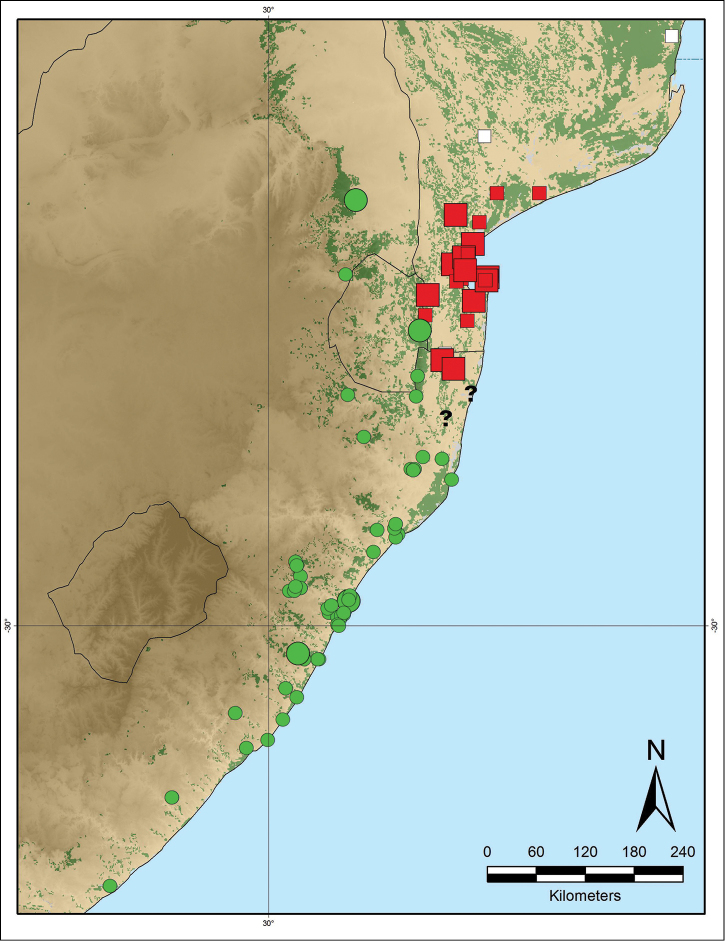
Distribution map of *Monanthotaxis maputensis* (red squares) and *Monanthotaxis caffra* (green circles). Large squares/circles denote flowering material of which the stamens were checked, small squares/circles are fruiting or sterile material or only seen from scans. White squares are aberrant fruiting collections of *Monanthotaxis maputensis*, question marks are fruiting specimens which have intermediate characters between the two species.

Figs 11 , 12 , Table 4

#### Type.

MOZAMBIQUE. Maputo, Moamba, Chinhanguanine, margem esquerda do rio Incomáti, 14 December 1979, *J. de Koning 7766* (holotype: WAG [WAG0349310!]; isotypes: MO [3880761!], LMA).

#### Diagnosis.

Closely related to *Monanthotaxis caffra* (Sond.) Verdc., but differs in having long filaments which occupy more than half of the total stamen length, while *Monanthotaxis caffra* has short filaments and the anther cells occupy more than half of the total stamen length. Further *Monanthotaxis maputensis* has shorter and less hairy leaves, the fruiting pedicels are more slender and the stipes are shorter than with *Monanthotaxis caffra*.

#### Description.

*Shrub*, scandent shrub or liana, to 10 m tall, diameter to 3 cm; young branches reddish brown, with scattered appressed or erect light-brown hairs 0.4 mm long, quickly glabrescent, old branches dark brown with (whitish) lenticels. **Leaves**: *petioles* 2–4 × 0.7–1 mm, grooved adaxially, indumentum as branches; *leaf lamina* 2.8–6.7(–8.1) × 1.5–3.3 cm, length:width ratio 1.6–2.7(–3.3), elliptic to ovate or slightly obovate, base cuneate to rounded, with slightly thickened margins, apex obtuse to acute, chartaceous/coriaceous, discolorous, upper side shiny dark-green, lower side glaucous to light green, upper side soon glabrescent, lower surface with appressed yellowish/light brown hairs 0.2 mm long, glabrescent, venation eucamptodromous, midrib yellowish or reddish, secondary veins 5–8, from base curving upwards, tertiary venation reticulate, raised adaxially and slightly abaxially, or not visible abaxially, often pellucid-punctate. **Inflorescences** leaf-opposed, composed of a solitary flower or a 2–3 flowered rhipidium; sympodial rachis 0–3 mm; *flowering pedicels* 6–14 × 0.3–0.4 mm, glabrescent; *lower bracts* broadly ovate, 1.5 × 1.4 mm; *upper bracts* broadly triangular to ovate, placed near middle of pedicel, 0.5 × 0.5 mm; *flower buds* ovate, dried greyish yellowish with yellow margins. **Flowers** bisexual; *receptacle* 2–3 mm in diameter, flat, with short brown hairs between the carpels and stamens; *sepals* 3, 0.5–0.7 × 1.5–2 mm, broadly ovate, with short reddish brown pubescence near the margins, apex obtuse; *petals* pale yellowish to yellow, inside drying reddish brown to purple. 6 in two whorls; *outer petals*, 2.5–4 × 3.2–4 mm, broad ovate, shortly pubescent with yellowish hairs on the outside, more densely pubescent near margins, inside densely pubescent at the apex; *inner petals*, 3.0–3.4 × 1.7–2.2 ovate to elliptic, outside and inside pubescent at apex; *stamens* 12–15 in one or two whorls, free, obconic to clavate, 0.8–1.2 mm long, filaments 0.4–0.8 mm long, anthers latrorse, theca 0.3–0.5 mm, connective glabrous, truncate, staminodes 0; *carpels* 10–13, 1.2–1.6 × 0.4–0.5 mm, subcylindric to ellipsoid, glabrous except for some hairs at the base, 1 (–2) ovules, stigma elongate, 0.3–0.4 mm long, grooved, glabrous. **Fruits**: *pedicels* 8–14 × 0.4–0.9 mm; *sepals* persistent; *stipes* 2.5–4.0 mm long, slightly to strongly longitudinally grooved, sparsely covered with appressed hairs when young; *monocarps* 1–10, globose to elliptic with 1(–2) seed, 7.5–15 × 5 mm, 2-seeded ones to 19 mm long, slightly constricted between the seeds, apex apiculate, apex 0.5 mm, rugulose to smooth, glabrous in ripe fruits, ripe fruits bright red. **Seeds** 5.5–8.0 × 4.5–6.6 mm, globose to ellipsoid, both ends rounded, ochre-brown, raphe not visible, ruminations lamelliform.

#### Distribution.

From just South of the border of South Africa and Mozambique north to 23 degrees in the province Inhambane in Mozambique. Figure 12.

#### Ecology.

Occurring in different types of thickets and forests on sandy soils, at 0–150 m altitude.

#### Phenology.

Flowers collected in November, December and February to April, fruits collected from March to September.

#### Conservation status.

Proposed IUCN Red List Category: **Least Concern**
(LC): EOO 43433 km^2^, AOO 128 km^2^. Within the distribution range it has been collected from more than 10 different localities and at least 3 nature reserves. Furthermore it is quite common in the coastal dunes of Mozambique, therefore we suggest the status of Least Concern for this species.

#### Etymology.

Named after the town and province Maputo, the center of the distribution and where most collections originate from.

#### Additional specimens examined (all paratypes, except when noted as excluded).

**MOZAMBIQUE. Gaza**: Macia-Messano, 28 Aug 1980, *A. Nuvunga 292* (WAG [WAG0065948]); 3 km from Gumbe, 26 May 1965, *Â. Pereira 456* (MO [2177512], SRGH; excluded as paratype); between João Belo and Lumane, 6 Mar 1941, *A.R. da Torre 2635* (LISC [LISC035251]). **Inhambane**: Massinga, Pomene, 10 km from Hotel to Rio Das Pedras, 16 Jul 1981, *J. de Koning 9063* (LMA, MO [3880770]; excluded as paratype). **Maputo**: Marracuene, 12 km from Vila Luísa to Manhiça, *M.F. Correia 578* (LISC, MO, SRGH); Ilha de Inhaca, 8 Jun 1970, *M.F. Correia 1690* (LISC); Lourençco Marques, Ilha da Inhaca, 20 Jun 1973, *M.F. Correia 2892* (M, MO); Maputo Reserve, 21 March 1976, *M.A. Diniz 102* (MO [2830862], WAG [WAG0053942]); Goba, 23 Feb 1955, A.W. Exell 565 (LISC); Ilha da Inhaca, 19 May 1984, *E.M.C. Groenendijk 1377* (MO [3877333], WAG [WAG0053945]); Ilha da Inhaca, 25 June 1984, *E.M.C. Groenendijk 1426* (MO [3877334], WAG [WAG0053936]); Ilha da Inhaca, 28 Sep 1984, *E.M.C. Groenendijk 1493* (MO [3877332], WAG [WAG0053944]); Ilha da Inhaca, 29 Jul 1985, *E.M.C. Groenendijk 1760* (LMU, WAG [WAG0067887], WAG [WAG0067888]); Maputo, 13 Feb 1947, *R.M. Hornby 2613* (L [L.1762399], P [P01954698], SRGH); Inhaca Island, 11 Aug 1980, *P.C.M. Jansen 7375* (WAG [WAG0053938]); 5 km from Matola-Gare, 13 Feb 1982, *P.C.M. Jansen 7808* (BR, G, LMA, WAG [WAG0243995], WAG [WAG0243996]); Rikatla, Nov 1917, *H.A. Junod 105* (G [G00308289]); Delagoa Bay, 1890, *H.A. Junod 255* (G [G00308287]); Delagoa Bay, 1893, *H.A. Junod 522* (G [G00308290]); nearby Bobole, 24 Nov 1978, *J. de Koning 7316* (LISC, LMA, MO [3878659]); between Boane and Catuane, 12 Jun 1979, *J. de Koning 7379* (K, LMU, MO [3880192], WAG [WAG0375988], WAG [WAG0375989]); 4 km from Catembe, 6 May 1981, *J. de Koning 8680* (BR [BR0000013186029], K, LISC, MO [3306014], SRGH); Manhica, 3.5 km from Palmeira, 15 Apr 1975, *A. Marques 2725* (WAG [WAG0053940]); Salamanga, 3 Jun 1948, *F.A. Mendonça 4480* (MO); Salamanga, 3 Jul 1948, *F.A. Mendonça 4493* (EA, WAG [WAG0053946]); Ilha da Inhaca, 3 Jul 1975, *A.R. Moura 56* (MA [MA376952], WAG [WAG0053941]); Lourenco Marques, 6 Jun 1946, *A. de A. Pimenta 8204* (LISC [2 sheets]); Ilha da Inhaca, 13 Feb 1965, *J.E. Rodrigues 329* (WAG [WAG0053934]); Delagoa Bay, 6 Jan 1898, *F.R.R. Schlechter 12006* (E [E00624364], G [G00308301], HBG, L [L 0188015], L [L 0188016], L [L 0188017], MO [3726269], P [P01954701], PH [PH00021144], US [553351]); Ilha da Inhaca, 6 Aug 1984, *D. Zunguze 781* (WAG [WAG0071706]). **SOUTH AFRICA. Kwazulu-Natal**: Ndumu Game Reserve, 16 Feb 1969, *E.S. Pooley 387* (E [E00624367], PRE); Usuthu Forest, 27 March 1969, 27 Mar 1969, *E.S. Pooley 450* (E [E00624366], NU, PRE); Ndumu to Maputa km 23, 7 Mar 1973, *F. White 10469* (FHO [00004050]); Tembe Elephant Park, 18 Nov 2000, *P.C. Zietsman 4264* (MO [5837895], NY [00642339]).

#### Discussion.

Most specimens of *Monanthotaxis maputensis* can be readily distinguished from the similar *Monanthotaxis caffra*, based on the vegetative and fruiting characters (table 4). These characters overlap in areas where the species occur in close vicinity. The differences in stamen morphology, however, remains clear, even in these areas. In South Africa, close to the border with Mozambique, *Monanthotaxis maputensis* is confined to sandy soils at low elevations (<200m), while *Monanthotaxis caffra* occurs there at higher elevations (>600m). Note however that *Monanthotaxis caffra* occurs at sea-level too, in South Africa (Figure 12). *Monanthotaxis caffra* and *Monanthotaxis maputensis* are also similar to *Monanthotaxis parvifolia* (Oliv.) Verdc., which can be distinguished by the rounded to cuneate leaf base (vs subcordate in *Monanthotaxis parvifolia*), the scattered soon glabrescent young branches (vs dense ferruginous pubescence in *Monanthotaxis parvifolia*). Other characteristics to distinguish *Monanthotaxis maputensis* from *Monanthotaxis parvifolia* are the shorter pedicels and shorter petioles in the former species, and fewer carpels in the latter (10–12 vs 12–17).

Two fruiting specimens in the northern range of the distribution (*A. Pereira 456* and *J. de Koning 9063*) were slightly aberrant. *A. Pereira 456* has slightly bigger leaves and thick stipes, while the specimen *J. de Koning 9063* is more hairy than other specimens and has erect hairs on the young branches. The specimens *K. Balkwill 2999* and *A.A. Balsinhas 3187* from Kwazulu-Natal had intermediate characters between *Monanthotaxis maputensis* and *Monanthotaxis caffra* and did not contain any stamens to verify the identification.

**Table 4.** Differences between *Monanthotaxis maputensis* and *Monanthotaxis caffra*.

	*Monanthotaxis maputensis*	*Monanthotaxis caffra*
Length of filament divided by total stamen length	0.5–0.7	0.2–0.3
Outer petal length	2.5–4.0 mm	5–7 mm
Leaf length	2.8–6.7 (–8.1) cm	5.5–10.8 cm
Leaf length:width ratio	1.6–2.7(–3.3)	2.6–3.4
Secondary veins	5–8	9–10
Fruiting pedicel width	0.4–0.9 mm	1.0–1.5 mm
Seed form	globose to ellipsoid	ellipsoid

### Monanthotaxis tripetala

Taxon classificationPlantaeMagnolialesAnnonaceae

P.H.Hoekstra sp. nov.

urn:lsid:ipni.org:names:60472953-2

Figs 5 , 13

#### Type.

CAMEROON. East Province, 15 km E of Dimako, village halfway Bertoua-Doumé, 11 June 1965, *A.J.M. Leeuwenberg 5828* (holotype: WAG (2 sheets, barcodes: WAG0110801!, WAG0110802!); isotypes: B [B100190273!], BR [BR0000014126253], C, EA!, K!, LISC, MO, P [P01967268!], PRE, YA).

#### Diagnosis.

Differs from other *Monanthotaxis* species in having bisexual flowers with three thick outer petals, and wanting or strongly reduced inner petals. The other species of *Monanthotaxis* with three petals or reduced inner petals have unisexual flowers, such as *Monanthotaxis diclina* (Sprague) Verdc. and *Monanthotaxis cauliflora* (Chipp) Verdc.

#### Description.

*Liana*, 3 m high, diameter 11 mm; young branches reddish brown, with very short appressed reddish brown hairs, old branches dark brown, cylindrical, glabrescent with few lenticels. **Leaves**: *petioles* 2–4 × 0.6–1.0 mm, indumentum as branches; *lamina* 4.2–12.3 × 1.8–4.9 cm, length:width ratio 1.8–2.8, oblong to elliptic, base rounded, with thick globose glands at base, apex acute to acuminate, subcoriaceous, discolorous, adaxially glossy dark green, abaxially dull greenish white, adaxially glabrescent, young with scattered appressed white hairs, midrib impressed, glabrescent with appressed yellowish hairs, abaxially glabrescent with scattered appressed white hairs, more dense and yellowish on midrib, venation eucamptodromous, secondary veins 7–9, from base curving upwards, tertiary venation scalariform, not visible adaxially. **Inflorescences** axillary, composed of a solitary flower or a 2-flowered rhipidium; sympodial rachis 0–6 mm with yellow appressed hairs; *flowering pedicels* 12–20 × 0.2–0.4 mm, with appressed yellowish hairs; *lower bract* wanting; *upper bract* in the upper half of the pedicel or wanting, lanceolate, 0.5 × 0.2 mm with dense hairs; *flower buds* rounded. **Flowers** bisexual; *sepals* 3, free, 0.5 × 0.8–1.0 mm, broadly ovate, apex obtuse, with dense yellow appressed hairs; *receptacle* 1.5 mm in diameter, flat; *petals* 3(–4) in one(-two) whorls; *outer petals*, 2.0–2.2 × 2.2 mm, ovate, with appressed yellowish hairs on the outside, and papillose near the apex on the inside; *inner petals* usually wanting, rarely a single strongly reduced petal present, 1.5 × 0.5–0.6 mm, elliptic, with scattered yellowish papillae on the outside and base of the inside; *stamens* 9–12 in 1 whorl, free, linear-clavate, 1.2 mm long, filaments 0.6 mm long, anther cells extrorse to lateral not hiding connective, glabrous, staminodes 0; *carpels* 9, 1.1–1.2 × 0.3–0.4 mm, subcylindric to ellipsoid, densely hairy, ovules 3–4 lateral, stigma subsessile 0.1–0.2 mm, glabrous. **Fruits**: unknown.

**Figure 13.**
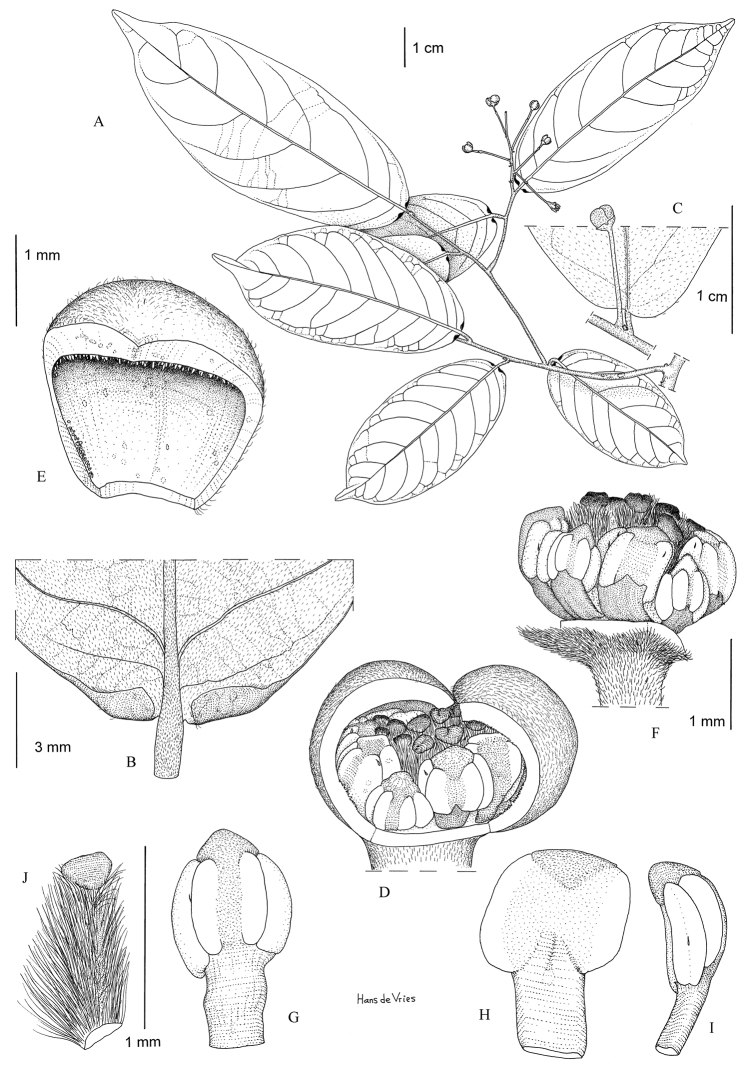
*Monanthotaxis tripetala* P.H.Hoekstra. **A–J** drawn from the type (Leeuwenberg 5828). **A** Habitus **B** Leaf base abaxially **C** Leaf base adaxially with inflorescence **D** Flower with one petal removed **E** Petal inside **F** Flower petals removed **G** Stamen outside **H** Stamen inside **I** Stamen lateral **J** Carpel. Illustration by H. de Vries.

#### Distribution.

Cameroon, East province and Gabon, Ogooué-Ivindo. Figure 5.

#### Ecology.

Evergreen Forest and Old secondary Forest, at 515–650 m altitude.

#### Phenology.

Flowers collected in June.

#### Conservation status.

Proposed IUCN Red List Category: **Endangered**
(ED): B2ab(iii), EOO 6370 km^2^, AOO 16 km^2^. This species is only known from 4 collections from 3 locations of which only one in a protected area. The other location (Belinga) is under threat of mining companies and the location in Cameroon is in an area with increasing human population growth, therefore we suggest the status of endangered.

#### Etymology.

Named for the three petals, one of the diagnostic characteristics of this species.

#### Additional specimens examined (all paratypes).

**GABON. Ogooué-Ivindo**: Station d’Ipassa, 22 Jun 1978, *J. Florence 1409* (P [P01985718]); Belinga, A. Moungazi 252 (P [P01982463]); Ipassa reserve, 0°30.39'N; 12°47.65'E, 7 Nov 2015, *J.J. Wieringa 8229* (WAG [WAG.1575731, WAG.1575732, WAG.1575733, WAG.1575734] & spirit collection [WAG0116914]).

#### Discussion.

This species can easily be distinguished from all other *Monanthotaxis* species by the small bisexual flowers with 3 outer petals and wanting to strongly reduced inner petals. Some other species exist, such as *Monanthotaxis cauliflora* (Chipp) Verdc., *Monanthotaxis diclina* (Sprague) Verdc. and *Monanthotaxis mortehanii* (De Wild.) Verdc., which have reduced inner petals, but those have unisexual flowers. Another probably new species from Cameroon which is currently still under study, also has completely wanting inner petals, but also that species has unisexual flowers.

The type specimen from Cameroon has reflexed and thickened edges at the leaf base (e.g. figure 13B), which is wanting in the specimens from Gabon, where only a slight depression can be seen next to the petiole insertion. This character is variable in other species as well, such as *Monanthotaxis schweinfurthii* (Engl. & Diels) Verdc. The very distinctive, small flowers with only 3(–4) petals in the Gabon specimens make these specimens belong to *Monanthotaxis tripetala*.

The specimen of Moungazi 252 has a gall on one of the branches, which has not been observed in any of the currently collected material of the *Monanthotaxis* species, while the collection of Wieringa 8229 has many brown velvety galls on one of the leaves (WAG spirit collection). The latter type of gall has been observed in other *Monanthotaxis* species in Central Africa.

### Monanthotaxis zenkeri

Taxon classificationPlantaeMagnolialesAnnonaceae

P.H.Hoekstra sp. nov.

urn:lsid:ipni.org:names:60472954-2

**Figure 14.**
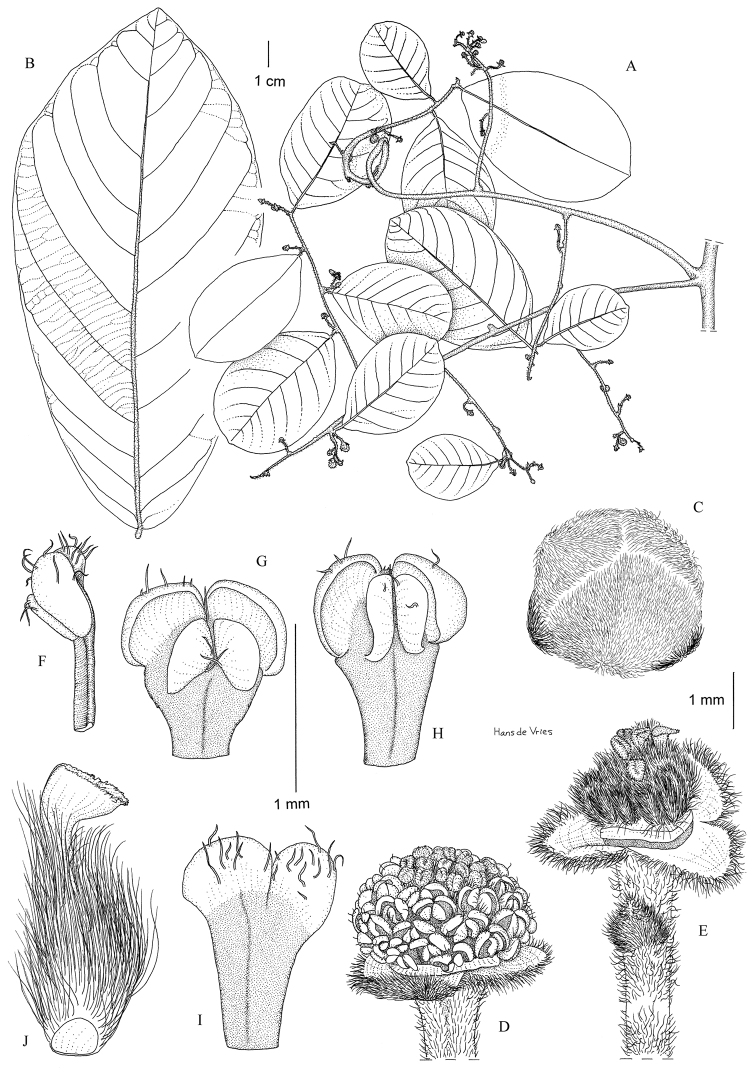
*Monanthotaxis zenkeri* P.H.Hoekstra. **A–J** drawn from the type (Zenker 3495a). **A** Habitus **B** Leaf underside **C** Flower bud **D** Flower with petals removed **E** Old flower **F** Stamen lateral **G–H** Stamen outside **I** Stamen inside **J** Carpel. Illustration by H. de Vries.

Figs 5 , 14 , Table 5

#### Type.

CAMEROON. South Province, Bipinde, probably October 1907, *G.A. Zenker 3495a* (holotype: G [G00308331!]; isotypes: BR [BR0000013211349!], E [E00624356!], HBG!, K!, L [L.1759466!], MO [3726267!]).

#### Diagnosis.

The only other two species of *Monanthotaxis* with the anther cells convergent apically hiding the connective are *Monanthotaxis bicornis* (Boutique) Verdc. and *Monanthotaxis filamentosa* (Diels) Verdc., it differs from both in having small flowers and further can be distinguished from the first by the almost glabrous leaves with cuneate base and having only 15 stamens, while *Monanthotaxis filamentosa* has long erect hairs on the stems and pedicels and has ovate to ovate-lanceolate flower buds with much longer petals.

#### Description.

Probably a *liana*. Young branches brown, densely covered with reddish brown, erect hairs 0.3–0.4 mm, old *branches* dark brown, cylindrical, glabrescent with few lenticels. **Leaves**: *petioles* 3–6 × 0.7–2.3 mm, indumentum as branches; *lamina* 4.7–20.1 × 2.3–9.5 cm, length:width ratio 2.0–2.3, obovate to elliptic-obovate, base rounded, with thickened margin at base, apex obtuse to acute, subcoriaceous to chartaceous, adaxially glabrescent, young with a few short erect yellow-brown hairs, midrib impressed with short erect yellow-brown hairs, lower surface with dense erect short yellow-brown hairs, venation eucamptodromous, secondary veins (8–)10–12, first straight halfway curving upwards, tertiary venation scalariform. **Inflorescences** axillary, a 1–3-flowered rhipidium; sympodial rachis 0–2 mm with dense short ascending to erect reddish brown hairs; *flowering pedicels* 4–6 × 0.4–0.5 mm, with dense short ascending to spreading hairs; *lower bract* strongly reduced or wanting; *upper bract* in the lower half of the pedicel, ovate, 0.6–0.8 × 0.5–0.8 mm with dense hairs; *flower buds* rounded; *sepals* 3, slightly connate at base, 1.1 × 1.5 mm, broadly ovate to broadly triangular, apex obtuse, with dense yellow appressed hairs; *receptacle* 1.5 mm in diameter, flat; *petals* 6 in two whorls; *outer petals*, 2.0–3.1 × 2.1–2.5 mm, ovate, short appressed yellowish hairs on the outside and inside along the margins, glabrous at base and center of the inside; *inner petals* 1.8–2.4 × 1.3–1.6 mm, rhombic, with dense short yellow hairs on the outside and apex of the inside; *stamens* 35 in 3 to 4 whorls, free, linear-obconic, 0.7–0.8 mm long, filaments 0.4 mm long, anther cells extrorse converging apically and hiding the connective, with hairs at edges of the anther cells, staminodes 0; *carpels* ca 16, 1.1–1.4 × 0.3 mm, subcylindric to ellipsoid, with dense hairs, ovules 4–5, lateral, stigma curved, elongate to subglobose, 0.2 mm long, glabrous, but long hairs at base of stigma. **Fruits**: unknown.

#### Distribution.

Cameroon, South province. Figure 5.

#### Ecology.

Forest.

#### Phenology.

Flowering in October.

#### Conservation status.

Proposed IUCN Red List Category: **Critically Endangered**
(CR): B2ab(iii), only known from the type collection, which was collected more than a hundred years ago in an unprotected area. Actually, the species may well be extinct already.

#### Etymology.

Named after G.A. Zenker, who collected many specimens of *Monanthotaxis* in Cameroon from the end of the 19^th^ and the beginning of the 20^th^ century. The types of seven species of *Monanthotaxis* were collected by him.

#### Discussion.

Sprague and Hutchinson (1916) noted that they had seen the specimen Zenker 3495a, but felt reluctant to describe it because of the immature flowers on the specimens in the Kew herbarium. However, the flowers are fully developed on the specimens of G and HBG where the flowers are open, some of which have lost the petals already.

*Monanthotaxis zenkeri* is one of three species of *Monanthotaxis* with anther cells converging at the apex of the stamen. It can be distinguished easily from the other two species by the small flowers and dense short erect hairs on the young branches and leaves (Table 5). Vegetatively, this species is quite similar to *Monanthotaxis diclina* (Sprague) Verdc. which also is densely covered with short reddish brown hairs. However, the flowers are very different, *Monanthotaxis diclina* has unisexual flowers, the female inflorescences are cauliflorous and the male inflorescences axillary. Furthermore, the stamens are very different as *Monanthotaxis diclina* has 6 stamen in one whorl with the anther cells latrorse and an external whorl of 12 staminodes.

**Table 5.** Differences between species of *Monanthotaxis* with anther cells converging on apex of stamen.

	*Monanthotaxis zenkeri*	*Monanthotaxis filamentosa*	*Monanthotaxis bicornis*
Outer petal length	2.0–3.1 mm	20–22 mm	5–8 mm
Nr of stamen whorls	3–4	3–4	2
Nr of stamens	35	34–46	15–18
Nr of carpels	16	9–14	6–9
Nr of ovules per carpel	4–5	7–10	3
Flower bud	globose	ovoid to lanceolate	rounded
Pedicel length	4–6 mm	0–8 mm	8–16 mm (Cameroon) 23–55 mm (Congo)
Indumentum on young branches	densely covered with erect hairs 0.3–0.4 mm	densely covered with erect hairs 1.2–2.0 mm	densely covered with appressed hairs 0.1–0.2 mm

## Supplementary Material

XML Treatment for Monanthotaxis atopostema

XML Treatment for Monanthotaxis aquila

XML Treatment for Monanthotaxis atewensis

XML Treatment for Monanthotaxis couvreurii

XML Treatment for Monanthotaxis filipes

XML Treatment for Monanthotaxis komorensis

XML Treatment for Monanthotaxis latistamina

XML Treatment for Monanthotaxis maputensis

XML Treatment for Monanthotaxis tripetala

XML Treatment for Monanthotaxis zenkeri
